# Frequency of Cardiac Valvulopathies in Patients With Marfan Syndrome: A Systematic Review and Meta-Analysis

**DOI:** 10.7759/cureus.54141

**Published:** 2024-02-13

**Authors:** Carlson Sama, Noah T Fongwen, Muchi Ditah Chobufo, Ademola Ajibade, Melissa Roberts, Mark Greathouse, Anthony Lyonga Ngonge, Ayowumi Adekolu, Yasmin S Hamirani

**Affiliations:** 1 Internal Medicine, West Virginia University School of Medicine, Morgantown, USA; 2 Public Health Sciences, Africa Centre for Disease Control and Prevention (Africa CDC), Addis Ababa, ETH; 3 Cardiology, West Virginia University School of Medicine, Morgantown, USA; 4 Cardiology, Morehouse School of Medicine, Atlanta, USA

**Keywords:** marfan syndrome, cardiac, prevalence, mitral valve, aortic valve, tricuspid valve, pulmonary valve, valvular disease

## Abstract

Marfan syndrome (MFS) is a progressive connective tissue disease with a broad range of clinical manifestations. We sought to establish the spectrum of structural valvular abnormalities as cardiovascular involvement has been identified as the most life-threatening aspect of the syndrome. This was a systematic review with a meta-analysis of studies indexed in Medline from the inception of the database to November 7, 2022. Using the random-effects model, separate Forest and Galbraith plots were generated for each valvular abnormality assessed. Heterogeneity was assessed using the *I^2^* statistics whilst funnel plots and Egger’s test were used to assess for publication bias. From a total of 35 studies, a random-effects meta-analysis approximated the pooled summary estimates for the prevalence of cardiac valve abnormalities as mitral valve prolapse 65% (95% CI: 57%-73%); mitral valve regurgitation 40% (95% CI: 29%-51%); aortic valve regurgitation 40% (95% CI: 28%-53%); tricuspid valve prolapse 35% (95% CI: 15%-55%); and tricuspid valve regurgitation 43% (95% CI: 8%-78%). Only one study reported on the involvement of the pulmonary valve (pulmonary valve prolapse was estimated at 5.3% (95% CI: 1.9%-11.1%) in a cohort of 114 patients with MFS). We believe this study provides a description of the structural valvular disease spectrum and may help inform providers and patients in understanding the clinical history of MFS in the current treatment era with its increased life expectancy.

## Introduction and background

Marfan syndrome (MFS) is a multi-systemic genetic disorder that primarily affects connective tissues [1]. It is named after Antoine Marfan, a French paediatrician who first described the condition in 1896 after noticing conspicuous features in a five-year-old girl [2]. The underlying aetiology of MFS usually involves mutations in FBN1, the gene encoding the extracellular matrix protein fibrillin-1, and is generally inherited in an autosomal dominant fashion with variable penetrance [1]. It is a relatively uncommon disorder with a reported incidence of 2-3 per 10,000 individuals, though some studies suggest this might be an underestimate [1,3,4].

MFS is often characterized by a wide range of clinical manifestations with cardinal features mainly involving the cardiovascular, skeletal, and ocular systems though adipose/muscle tissue, pulmonary, cutaneous, and central nervous systems may also be affected [1,5-7]. As initially outlined by McKusick [8] and Murdoch et al. [9], involvement of the cardiovascular system is the major cause of death with aortic dissection or rupture being the most common cause of these premature deaths, however, cardiac valve disease also contributes significantly to the observed high morbidity and early mortality, especially during childhood [3,10]. As percutaneous techniques allow valve interventions in a broader population of patients, it seems timely to review the prevalence and prognosis of valve disease in patients with MFS. In this review, we try to establish the different frequencies at which these valves are defective in MFS in a bid to establish a baseline in this era of improved valvular interventions.

Materials and methods

This paper is written and reported in accordance with the Preferred Reporting Items for Systematic Reviews and Meta-Analysis Protocols (PRISMA-P) 2020 Guidelines [11].

Inclusion Criteria

We included observational studies that consisted of patients with a formal diagnosis of MFS (diagnosed either by genetic studies or the Ghent nosology criteria [12,13]). Our primary outcome of interest was structural cardiac valve disorders diagnosed by transthoracic echocardiography on initial presentation. No language or location restriction was applied.

Exclusion Criteria

We excluded studies lacking rates of valvulopathy in patients with MFS, with the absence of data to compute the relevant effect sizes. Studies in which diagnosis of valvulopathy was made by any other means than echocardiography (e.g., by auscultation) were also excluded.

Search Strategy and Identification of Studies

A systematic search strategy combining relevant medical subject headings (MeSH) such as “mitral valve”, “tricuspid valve”, “pulmonary valve” and “aortic valve” with the appropriate Boolean operators in combination with “marfan’s syndrome” was used to search MEDLINE for eligible studies from the inception of the database to November 7, 2022. No restrictions were placed when running the search and articles returned were saved to the Zotero software (Corporation for Digital Scholarship, Vienna, USA). The titles and abstracts of the articles were reviewed against eligibility criteria, and the full text of the articles meeting inclusion was acquired for review. We further hand-searched the reference list of eligible full-text articles to obtain additional data.

Study Records, Screening, and Data Extraction

Retained data were uploaded to the Rayyan software (Qatar Computing Research Institute, Qatar) to facilitate online collaboration between investigators. A standardised pretested questionnaire was also drafted by the investigators to orient the screening of titles and abstracts. Two independent reviewers (CS and NTF) rigorously reviewed the full text of all potentially eligible studies with disagreements resolved by a third reviewer (CMD). Data was extracted onto a pretested Excel spreadsheet (produced on Microsoft Excel v.2013, Microsoft® Corp., Redmond, WA).

Assessment of Methodological Quality and Risk of Bias

The quality of the included studies was independently scored by two reviewers (CS and NTF). The Strengthening the Reporting of Observational Studies in Epidemiology (STROBE) checklist [14] for observational studies was used to evaluate the quality of reporting in each paper. The Risk of Bias Tool for Prevalence Studies developed by Hoy and collaborators [15] was used to assess the risk of bias for each study.

Data Synthesis, Analysis, and Assessment of Heterogeneity

The information on the data extraction sheet was exported to the Stata software (Stata Corp V.17, Texas, USA), and analysed accordingly. After stabilising the variance of individual studies using the Freeman-Tukey double arc-sine transformation [16], heterogeneity was assessed using the χ^2 ^test on Cochrane’s Q statistic, Galbraith plots, as well as the I^2^ [17] and the tau-squared (τ^2^) statistics. The random effects meta-analysis models were preferentially reported over fixed-effects models to obtain an overall pooled summary estimate of prevalence rates and proportions across studies. Where substantial heterogeneity was detected, meta-regressions and subgroup stratified analyses were done to detect its possible sources according to important study characteristics. Cohen’s κ coefficient was used to assess inter-rater agreement for study inclusion [18]. Funnel plots, as well as Egger’s weighted regression methods, were used to assess for possible publication bias [19]. A p-value < 0.1 was considered indicative of statistically significant publication bias.

## Review

Narrative synthesis

**Figure 1:**
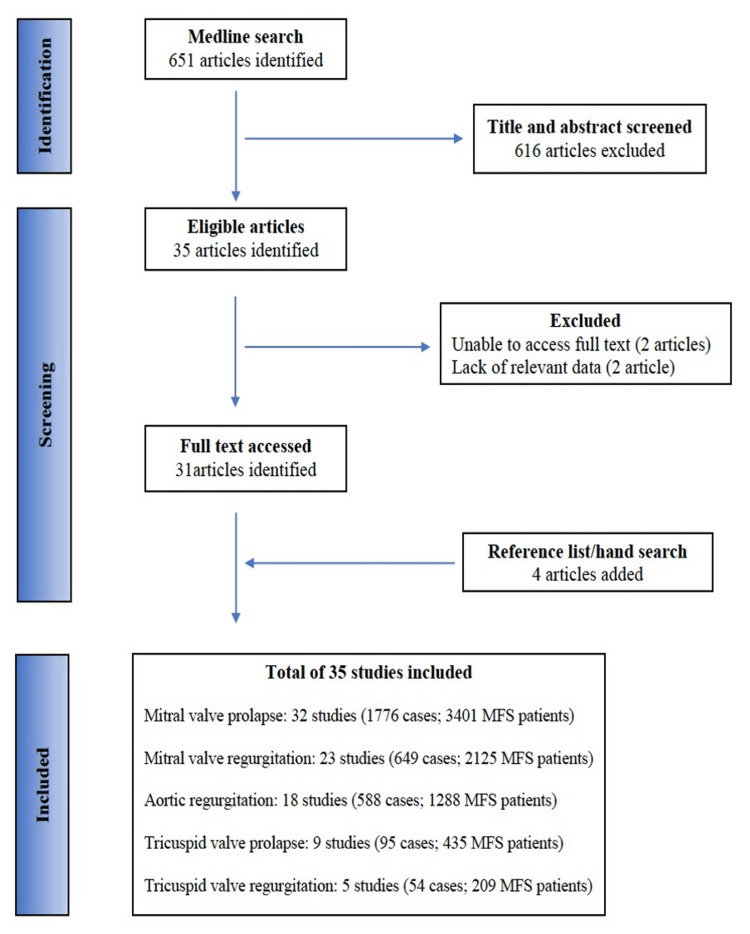
PRISMA search flow diagram for inclusion of articles in the meta-analysis PRISMA: Preferred Reporting Items for Systematic Reviews and Meta-Analysis, MFS: Marfan syndrome

A total of 35 studies published between 1975 and 2021 were included. Studies were stratified according to the WHO regions [20] and the majority of them were conducted in the Americas, specifically the USA. Figure 1 details search results and retained articles with the corresponding frequencies of valve abnormalities and sample sizes. The general characteristics of included studies for each valvular abnormality have been extensively presented in their corresponding tables (Tables 1-5). Over half (51.4%) of the included studies had a low risk of bias, with the remainder at moderate risk (48.6%). Of note, only one study reported on the involvement of the pulmonary valve in patients with MFS and thus was not included in a metanalysis but narratively reported here; the prevalence of pulmonary valve prolapse was estimated at 5.3% (95% CI: 1.9%-11.1%) in a cohort of 114 patients [21].

**Table 1:** Characteristics of studies reporting on mitral valve prolapse (MVP) Africa Region = AFR; The Americas = AMR; South-East Asian Region = SEAR; European Region = EUR; Eastern Mediterranean Region = EMR; Western Pacific Region = WPR SD: standard deviation, NR: not reported, MFS: Marfan syndrome Data in "Males" column is in the format: *n (*%)

Author	Year of publication	Frequency of MVP	Sample size	Timing of data collection	Mean age (years)	Mean age SD (years)	Males,* n* (%)	Diagnosis of MFS	Risk of bias	Country	WHO region
De Backer et al. [22]	2006	51	77	prospective	25	10.9	36 (46.8%)	Ghent criteria	low	USA/Belgium	AMR/EUR
Fan et al. [23]	2021	53	112	retrospective	24	11.45	60 (53.6)	Ghent criteria	moderate	Taiwan	WPR
Brown et al. [24]	1975	13	35	prospective	20.9	13.6	23 (65.7)	Ghent criteria	low	USA	AMR
Roman et al. [25]	2017	422	789	retrospective	23.1	9.1	421 (53.4)	Ghent criteria	moderate	USA	AMR
Gillinov et al. [26]	1997	20	26	prospective	10.3	1	18 (69.2)	Ghent criteria	moderate	USA	AMR
Bruno et al. [27]	1984	23	29	prospective	23.7	NR	23 (79.3)	Ghent criteria	low	Italy	EUR
Lacro et al. [28]	2013	230	608	prospective	11.2	6.4	366 (60.2)	Ghent criteria	low	USA	AMR
Attias et al. [29]	2009	105	232	prospective	30.2	16.2	119 (51.3)	Genetic studies	low	France	EUR
Wozniak-Mielczarek et al. [30]	2018	55	101	prospective	23.8	15.32	55 (54.5)	Ghent criteria	moderate	Poland	EUR
Ozdemir et al. [31]	2011	11	11	prospective	9.6	4.2	7 (63.6)	Ghent criteria	low	Turkey	EUR
Gu et al. [32]	2015	15	73	retrospective	32	12	52 (71.2)	Ghent criteria	moderate	China	WPR
Rybczynski et al. [33]	2010	82	204	prospective	31.2	16.4	108 (52.9)	Ghent criteria	moderate	Germany	EUR
Mueller et al. [34]	2013	26	82	prospective	9.01	5.7	46 (56.1)	Ghent criteria or Genetic studies	low	Germany	EUR
Seo et al. [35]	2016	8	8	retrospective	0.5	0.6	2 (25)	Ghent criteria	moderate	South Korea	WPR
Marsalese et al. [36]	1989	27	44	retrospective				Ghent criteria	moderate	USA	AMR
Tayel et al. [37]	1991	13	15	retrospective	8.9	2.6	8 (53.3)	Ghent criteria	moderate	USA	AMR
Kunkala et al. [38]	2013	166	234	retrospective	32	13	93 (39.7)	Ghent criteria	moderate	USA/Germany	AMR/EUR
Pyeritz et al. [39]	1983	113	166	retrospective	11.5	0.6	84 (50.6)	Ghent criteria	moderate	USA	AMR
van Karnebeek et al. [40]	2001	46	52	prospective	15.5	NR	25 (48.1)	Ghent criteria and Genetic studies	low	Netherlands	EUR
Veldhoen et al. [41]	2013	20	31	prospective	11.5	NR	16 (51.6)	Ghent criteria	low	Germany	EUR
Lopez et al. [42]	2005	11	21	prospective	9.18	4.5	14 (66.7)	Ghent criteria	moderate	Brazil	AMR
Geva et al. [43]	1987	25	25	retrospective	8.1	4.8	21 (84)	Ghent criteria	moderate	Israel	EUR
Sisk et al. [44]	1983	9	15	prospective	1.6	1.04	5 (33.3)	Ghent criteria	low	USA	AMR
Hirata et al. [45]	1992	22	24	prospective	28.2	8.6	16 (66.7)	Ghent criteria	low	USA	AMR
Figueiredo et al. [46]	2001	18	20	retrospective	NR	NR	NR	Ghent criteria	moderate	Portugal	EUR
Come et al. [47]	1983	35	61	prospective	28.4	12.5	36 (59)	Ghent criteria	low	USA	AMR
Freed et al. [48]	1977	7	11	prospective	29	12.2	6 (54.5)	Ghent criteria	low	USA	AMR
Pan et al. [49]	1985	12	12	prospective	17.7	12.3	5 (41.7)	Ghent criteria	low	China	WPR
Yetman et al. [50]	2003	34	70	prospective	NR		34 (48.6)	Ghent criteria	low	USA/Canada	AMR
Taub et al. [51]	2009	25	90	prospective	29	14	45 (50)	Ghent criteria or Genetic studies	low	USA	AMR
Geva et al. [52]	1990	9	9	prospective	0.225	0.34	5 (55.6)	Ghent criteria	low	NR	AMR
Espınola-Zavaleta et al. [21]	2010	70	114	prospective	7.8	4.2	53 (46.5)	Ghent criteria	low	NR	AMR

**Table 2:** Characteristics of studies reporting on mitral valve regurgitation (MVR) Africa Region = AFR; The Americas = AMR; South-East Asian Region = SEAR; European Region = EUR; Eastern Mediterranean Region = EMR; Western Pacific Region = WPR SD: standard deviation, NR: not reported, MFS: Marfan syndrome Data in "Males" column is in the format: n (%)

Author	Year of publication	Frequency of MVR	Sample size	Timing of data collection	Mean age (years)	Mean age SD (years)	Males,* n* (%)	Diagnosis of MFS	Risk of bias	Country	WHO region
De Backer et al. [22]	2006	2	77	prospective	25	10.9	36 (46.8)	Ghent criteria	low	USA/Belgium	AMR/EUR
Fan et al. [23]	2021	70	112	retrospective	24	11.5	60 (53.6)	Ghent criteria	moderate	Taiwan	WPR
Forteza et al. [53]	2009	6	37	retrospective	30	10	25 (67.6)	Ghent criteria	moderate	Spain	EUR
Svensson et al. [54]	2007	70	122	retrospective	39	12.7	82 (67.2)	Ghent criteria	moderate	USA	AMR
Gillinov et al. [26]	1997	10	26	prospective	10.3	1	18 (69.2)	Ghent criteria	moderate	USA	AMR
Lacro et al. [28]	2013	108	608	prospective	11.2	6.4	366 (60.2)	Ghent criteria	low	USA	AMR
Attias et al. [29]	2009	129	230	prospective	30.2	16.2	119 (51.7)	NR	low	France	EUR
Wozniak-Mielczarek et al. [30]	2018	64	101	prospective	23.76	15.3	55 (54.5)	Ghent criteria	moderate	Poland	EUR
Ozdemir et al. [31]	2011	9	11	prospective	9.6	4.2	7 (63.6)	Ghent criteria	low	Turkey	EUR
Gu et al. [32]	2015	15	73	retrospective	32	12	52 (71.2)	Ghent criteria	moderate	China	WPR
Rybczynski et al. [33]	2010	25	204	prospective	31.2	16.4	108 (52.9)	Ghent criteria	moderate	Germany	EUR
Mueller et al. [34]	2013	18	82	prospective	9.01	5.7	46 (56.1)	Ghent criteria or Genetic studies	low	Germany	EUR
Seo et al. [35]	2016	8	8	retrospective	0.5	0.6	2 (25)	Ghent criteria	moderate	South Korea	WPR
Tayel et al. [37]	1991	8	15	retrospective	12.5	2.6	8 (53.3)	Ghent criteria	moderate	USA	AMR
Pyeritz et al. [39]	1983	15	166	retrospective	11.5	0.6	84 (50.6)	Ghent criteria	moderate	USA	AMR
van Karnebeek et al. [40]	2001	25	52	prospective	15.5		25 (48.1)	Ghent criteria and Genetic studies	low	Netherlands	EUR
Veldhoen et al. [41]	2013	16	31	prospective	11.5		16 (51.6)	Ghent criteria	low	Germany	EUR
Lopez et al. [42]	2005	3	21	prospective	9.18	4.5	14 (66.7)	Ghent criteria	moderate	Brazil	AMR
Sisk et al. [44]	1983	5	15	prospective	1.6	1.04	5 (33.3)	Ghent criteria	low	USA	AMR
Hirata et al. [45]	1992	12	24	prospective	28.2	8.6	16 (66.7)	Ghent criteria	moderate	USA	AMR
Davis et al. [55]	1978	8	31	NR	NR	NR	NR	NR	moderate	USA	AMR
Yetman et al. [50]	2003	15	70	prospective			34 (48.6)	Ghent criteria	low	USA/Canada	AMR
Geva et al. [52]	1990	8	9	prospective	0.225	0.34	5 (55.6)	Ghent criteria	low	USA	AMR

**Table 3:** Characteristics of studies reporting on aortic valve regurgitation (AVR) Africa Region = AFR; The Americas = AMR; South-East Asian Region = SEAR; European Region = EUR; Eastern Mediterranean Region = EMR; Western Pacific Region = WPR SD: standard deviation, NR: not reported, MFS: Marfan syndrome Data in "Males" column is in the format: n (%)

Author	Year of publication	Frequency of AVR	Sample size	Timing of data collection	Mean age (years)	Mean age SD (years)	Males,* n* (%)	Diagnosis of MFS	Risk of bias	Country	WHO region
Forteza et al. [53]	2009	28	37	retrospective	30	10	25 (67.6)	Ghent criteria	moderate	Spain	EUR
Svensson et al. [54]	2007	80	122	retrospective	39	12.7	82 (67.2)	Ghent criteria	moderate	USA	AMR
Roman et al. [25]	2017	279	606	retrospective	23.1	9.1	421 (69.5)	Ghent criteria	moderate	USA	AMR
Gillinov et al. [26]	1997	9	26	prospective	10.3	1	18 (69.2)	Ghent criteria	moderate	USA	AMR
Wozniak-Mielczarek et al. [30]	2018	26	84	prospective	23.8	15.32	55 (65.5)	Ghent criteria	moderate	Poland	EUR
Ozdemir et al. [31]	2011	2	11	prospective	9.6	4.2	7 (63.6)	Ghent criteria	low	Turkey	EUR
Gu et al. [32]	2015	63	73	retrospective	32	12	52 (71.2)	Ghent criteria	moderate	China	WPR
Mueller et al. [34]	2013	8	82	prospective	9	5.7	46 (56.1)	Ghent criteria or Genetic studies	moderate	Germany	EUR
Seo et al. [35]	2016	3	8	retrospective	0.5	0.6	2 (25)	Ghent criteria	moderate	South Korea	WPR
Marsalese et al. [36]	1989	19	44	retrospective	25			Ghent criteria	moderate	USA	AMR
Tayel et al. [37]	1991	1	15	retrospective	8.9	2.6	8 (53.3)	Ghent criteria	moderate	USA	AMR
van Karnebeek et al. [40]	2001	13	52	prospective	15.5	NR	25 (48.1)	Ghent criteria and Genetic studies	low	Netherlands	EUR
Lopez et al. [42]	2005	3	21	prospective	9.2	4.5	14 (66.7)	Ghent criteria	moderate	Brazil	AMR
Geva et al. [43]	1987	7	25	retrospective	8.1	4.8	21 (84)	Ghent criteria	moderate	Israel	EUR
Hirata et al. [45]	1992	6	24	prospective	28.2	8.6	16 (66.7)	Ghent criteria	moderate	USA	AMR
Nguyen et al. [56]	1997	14	18	prospective	29	NR	NR	NR	moderate	USA	AMR
Davis et al. [55]	1978	26	31		NR	NR	NR	NR	moderate	USA	AMR
Geva et al. [52]	1990	1	9	prospective	0.2	0.34	5 (55.6)	Ghent criteria	low	USA	AMR

**Table 4:** Characteristics of studies reporting on the tricuspid valve prolapse (TVP) Africa Region = AFR; The Americas = AMR; South-East Asian Region = SEAR; European Region = EUR; Eastern Mediterranean Region = EMR; Western Pacific Region = WPR SD: standard deviation, NR: not reported, MFS: Marfan syndrome Data in "Males" column is in the format: n (%)

Author	Year of publication	Frequency of TVP	Sample size	Timing of data collection	Mean age (years)	Mean age SD (years)	Males,* n* (%)	Diagnosis of MFS	Risk of bias	Country	WHO region
Wozniak-Mielczarek et al. [30]	2018	6	101	prospective	23.76	15.3	55 (54.5)	Ghent criteria	moderate	Poland	EUR
Ozdemir et al. [31]	2011	7	11	prospective	9.6	4.2	7 (63.6)	Ghent criteria	low	Turkey	EUR
Gu et al. [32]	2015	8	73	retrospective	32	12	52 (71.2)	Ghent criteria	moderate	China	WPR
Mueller et al. [34]	2013	14	82	prospective	9.01	5.7	46 (56.1)	Ghent criteria or Genetic studies	low	Germany	EUR
Seo et al. [35]	2016	6	8	retrospective	0.5	0.6	2 (25)	Ghent criteria	moderate	South Korea	WPR
Geva et al. [43]	1987	1	25	retrospective	8.1	4.8	21 (84)	Ghent criteria	moderate	Israel	EUR
Pan et al. [49]	1985	4	12	prospective	17.7	12.3	5 (41.7)	Ghent criteria	low	China	WPR
Geva et al. [52]	1990	8	9	prospective	0.2	0.3	5 (55.6)	Ghent criteria	low	NR	AMR
Espinola-Zavaleta et al. [21]	2010	41	114	prospective	7.8	4.2	53 (46.5)	Ghent criteria	low	NR	AMR

**Table 5:** Characteristics of studies reporting on tricuspid valve regurgitation (TVR) Africa Region = AFR; The Americas = AMR; South-East Asian Region = SEAR; European Region = EUR; Eastern Mediterranean Region = EMR; Western Pacific Region = WPR SD: standard deviation, NR: not reported, MFS: Marfan syndrome Data in "Males" column is in the format: n (%)

Author	Year of publication	Frequency of TVR	Sample size	Timing of data collection	Mean age (years)	Mean age SD (years)	Males,* n* (%)	Diagnosis of MFS	Risk of bias	Country	WHO region
Forteza et al. [53]	2009	3	37	retrospective	30	10	25 (67.6)	Ghent criteria	moderate	Spain	EUR
Gu et al. [32]	2015	7	73	retrospective	32	12	52 (71.2)	Ghent criteria	moderate	China	WPR
Mueller et al. [34]	2013	30	82	prospective	9.01	5.7	46 (56.1)	Ghent criteria or Genetic studies	low	Germany	EUR
Seo et al. [35]	2016	8	8	retrospective	0.5	0.6	2 (25)	Ghent criteria	moderate	South Korea	WPR
Geva et al. [52]	1990	6	9	prospective	0.225	0.34	5 (55.6)	Ghent criteria	low	USA	AMR

Meta-analysis

**Figure 2:**
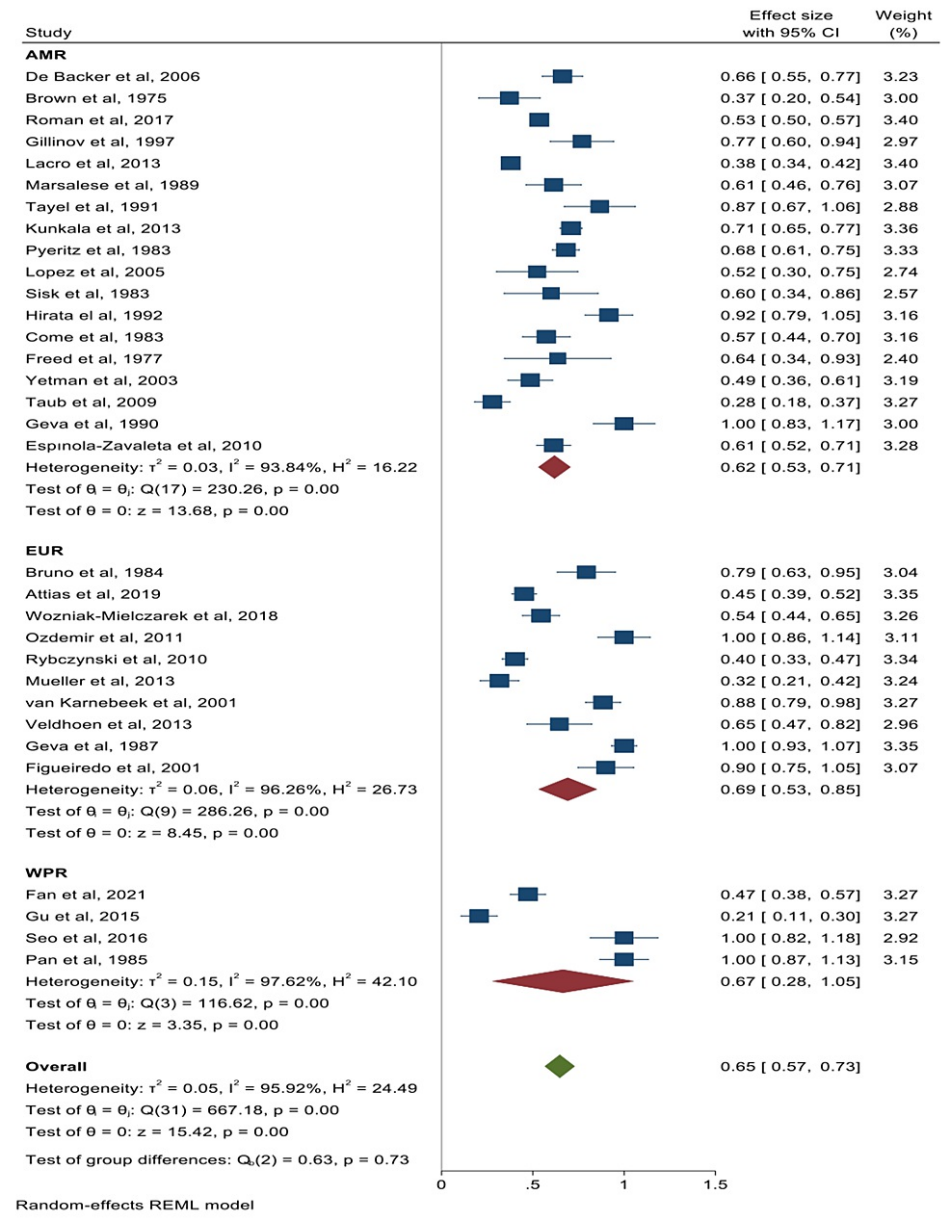
Forest plot for studies reporting on the prevalence of mitral valve prolapse De Backer et al. [22], Fan et al. [23], Brown et al. [24], Roman et al. [25], Gillinov et al. [26], Bruno et al. [27], Lacro et al. [28], Attias et al. [29], Wozniak-Mielczarek et al. [30], Ozdemir et al. [31], Gu et al. [32], Rybczynski et al. [33], Mueller et al. [34], Seo et al. [35], Marsalese et al. [36], Tayel et al. [37], Kunkala et al. [38], Pyeritz et al. [39], van Karnebeek et al. [40], Veldhoen et al. [41], Lopez et al. [42], Geva et al. [43], Sisk et al. [44], Hirata et al. [45], Figueiredo et al. [46], Come et al. [47], Freed et al. [48], Pan et al. [49], Yetman et al. [50], Taub et al. [51], Geva et al. [52], Espınola-Zavaleta et al. [21] The Americas = AMR; European Region = EUR; Western Pacific Region = WPR

**Figure 3:**
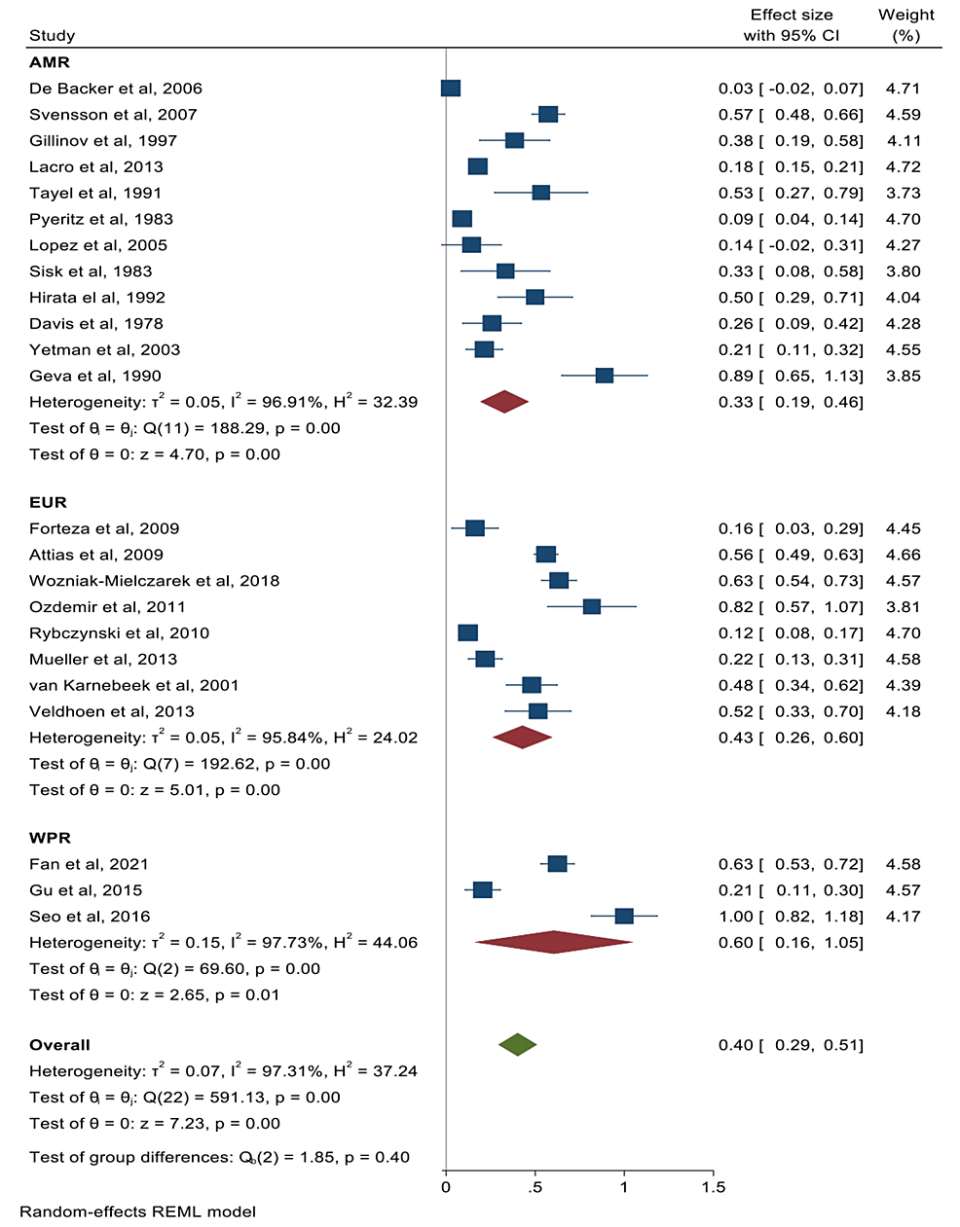
Forest plot for studies reporting on the prevalence of mitral valve regurgitation De Backer et al. [22], Fan et al. [23], Forteza et al. [53], Svensson et al. [54], Gillinov et al. [26], Lacro et al. [28], Attias et al. [29], Wozniak-Mielczarek et al. [30], Ozdemir et al. [31], Gu et al. [32], Rybczynski et al. [33], Mueller et al. [34], Seo et al. [35], Tayel et al. [37], Pyeritz et al. [39], van Karnebeek et al. [40], Veldhoen et al. [41], Lopez et al. [42], Sisk et al. [44], Hirata et al. [45], Davis et al. [55], Yetman et al. [50], Geva et al. [52] The Americas = AMR; European Region = EUR; Western Pacific Region = WPR

**Figure 4:**
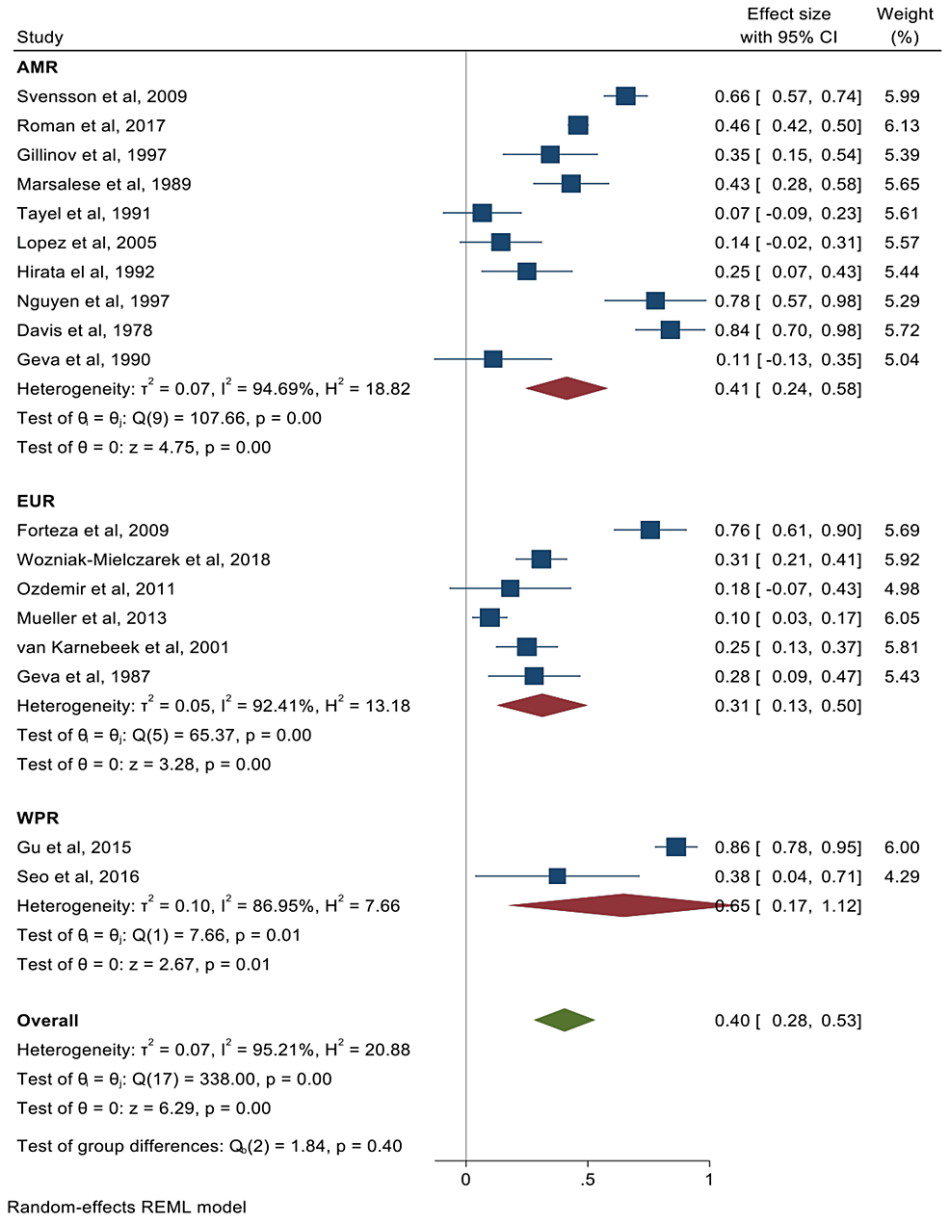
Forest plot for studies reporting on the prevalence of aortic valve regurgitation Forteza et al. [53], Svensson et al. [54], Roman et al. [25], Gillinov et al. [26], Wozniak-Mielczarek et al. [30], Ozdemir et al. [31], Gu et al. [32], Mueller et al. [34], Seo et al. [35], Marsalese et al. [36], Tayel et al. [37], van Karnebeek et al. [40], Lopez et al. [42], Geva et al. [43], Hirata et al. [45], Nguyen et al. [56], Davis et al. [55], Geva et al. [52] The Americas = AMR; European Region = EUR; Western Pacific Region = WPR

**Figure 5:**
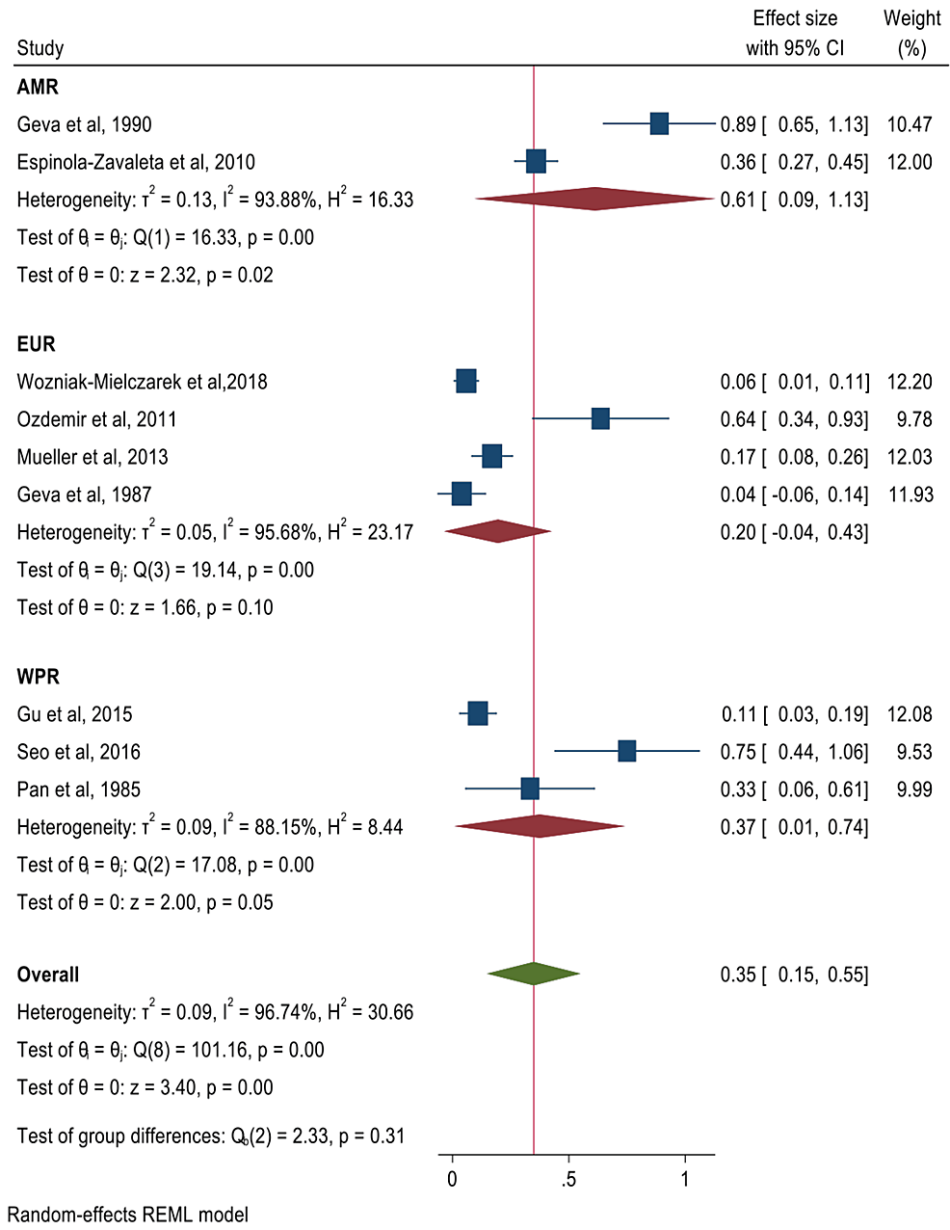
Forest plot for studies reporting on the prevalence of tricuspid valve prolapse Wozniak-Mielczarek et al. [30], Ozdemir et al. [31], Gu et al. [32], Mueller et al. [34], Seo et al. [35], Geva et al. [43], Pan et al. [49], Geva et al. [52], Espinola-Zavaleta et al. [21] The Americas = AMR; European Region = EUR; Western Pacific Region = WPR

**Figure 6:**
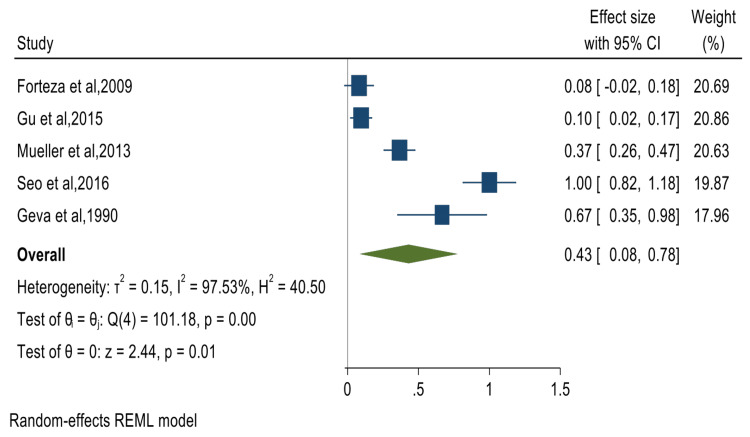
Forest plot for studies reporting on the prevalence of tricuspid valve regurgitation Forteza et al. [53], Gu et al. [32], Mueller et al. [34], Seo et al. [35], Geva et al. [52]

The pooled summary effects for the prevalence of cardiac valve abnormalities and their corresponding forest plots were mitral valve prolapse (MVP) 65% (95% CI: 57%-73%) (Figure 2); mitral valve regurgitation 40% (95% CI: 29%-51%) (Figure 3); aortic valve regurgitation 40% (95% CI: 28%-53%) (Figure 4); tricuspid valve prolapse 35% (95% CI: 15%-55%) (Figure 5); and tricuspid valve regurgitation 43% (95% CI: 8%-78%) (Figure 6). There was significant heterogeneity between studies (I2 ≥75%). Except for studies reporting on the rates of MVP for which heterogeneity could be explained by mean age [-0.0094 (95% CI: -0.0176 to -0.0013), p-value 0.024], the rest of the observed heterogeneity in the other primary outcomes was not explained by study design, study quality, geographical location, mean age, or sample size on sub-group meta-analysis and univariate meta-regression.

Discussion

This study describes the echocardiographic valvular examination of the largest cohort of MFS patients. We found that aortic regurgitation (AR) occurs in about 40% of patients with MFS. Whilst it may occur as an isolated finding, it is often preceded by aortic root dilation (ARD). Thus, the observed high prevalence of AR in this study suggests an even higher occurrence of ARD in patients with MFS. This carries clinical significance as ARD and its deleterious sequelae of aortic dissection and rupture is one of the most important prognostic features and accounts for most of the premature mortality among patients with MFS without appropriate treatment [9,40]. Thus, early management interventions in patients with clinically significant ARD can reduce the incidence of AR, and therefore, ultimately reducing adverse outcomes.

We found the prevalence of MVP to be homogeneous across regions; 62% in the Americas, 69% in Europe, and 67% in the Western Pacific Region. The homogeneity across these different independent regions suggests this might be the true prevalence of MVP in a population of MFS patients. Overall, our pooled estimate of 65% is significantly higher than the reported 2-3% prevalence of MVP in the general population [40,50,57]. Our findings also indicate that MVP is the most common valvular abnormality occurring in patients with MFS. This has implications as it is known that MVP carries a definitive risk of progression to complications such as mitral regurgitation (MR) which had a prevalence of 40% in this study. Of note, the other downstream effects of these mitral valvulopathies include atrial fibrillation, heart failure, stroke, and infective endocarditis [34,57-59], and in the worst case, rupture of the chordae tendineae leading to acute MR and heart failure that may precipitate the need for urgent surgery [39,52,60,61]. Important to further highlight that these complications occur at a higher frequency in patients with MFS than those without; for example, it has been observed that those with MFS and MVP have a greater risk of endocarditis (13.4% at age 60 years) than persons from the general population who have MVP, in whom endocarditis develops only in about 0.48% [33,62]. This suggests poorer cardiovascular outcomes in patients with MFS as opposed to those without MFS for a similar valvulopathy, thus the need for heightened clinical follow-up amongst MFS patients with cardiovascular involvement cannot be overemphasised.

Most published reports concerning the evaluation and treatment of patients with MFS have generally excluded descriptions of the tricuspid valve. We however noted that the occurrence of tricuspid valve abnormalities in this population is quite significant with a prevalence of 35% for tricuspid prolapse and 43% for tricuspid regurgitation. Gu et al. [32] also noted that all their MFS patients with tricuspid valve dysfunction also had concomitant mitral valve dysfunction, thus suggesting the evaluation of MFS patients should also include a detailed evaluation of the tricuspid valve, especially in the paediatric population where it has been reported to be more prevalent and is associated with significant cardiovascular morbidity [34,52].

Of note, in our cohort of patients with MFS, we did not find any report of valvular stenosis. In part, this might be related to the MFS pathophysiology of fibro-myxomatous degeneration of valvular tissues secondary to underlying extracellular matrix derangements leading to a more floppy valve as opposed to a rigid and stenotic lesion [63]. Furthermore, though MFS is a diffuse disease process that can potentially affect any or all cardiac valve tissues, only one study reported on the involvement of the pulmonary valve in patients with MFS and thus was not included in a metanalysis but narratively reported here; the prevalence of pulmonary valve prolapse was estimated at 5.3% in a cohort of 114 patients [21]. We hypothesize that the small sample size of this single study may not be a true reflection of the occurrence of pulmonary valvulopathies in the general MFS population. It is however unclear if the description of the valve is simply being underreported or is just not evaluated, though a combination of the two seems likely. Regardless, our findings suggest that pulmonary valvulopathy as well as valvular stenosis in patients with MFS is quite uncommon.

Overall, it has been noted that echocardiography improves the routine detection of underlying cardiovascular disease in patients with MFS when compared to physical examination (auscultation) [47]. Given that the valvular damage caused by MFS usually manifests silently in the early stages of childhood and is progressive throughout life, serial echocardiography follow-up can often facilitate elective surgery and is imperative [21], especially in the current treatment era where prophylactic surgery has shown to be crucial in preventing MFS-related valvular complications, with a resultant improvement in life expectancy [23,53].

There are limitations to this study, firstly, the use of a single database might have increased the likelihood of missing out on some studies. The relatively small sample size in some individual studies may lead to unstable effect sizes, thus affecting its power and increasing the margin of error. In addition, we also observed significant heterogeneity when pooling some studies which could not be explained by tested stratified analysis. Further, it remains unclear how the observed heterogeneity and different diagnostic criteria for MFS (genetic vs. clinical) may have affected our findings, especially as there were some revisions to the clinical criteria over the study period. Similarly, echocardiographic investigations were sampled using different methods in different studies without clearly defined criteria for each valvular disorder, thus a chance for over/under-estimating valvulopathies exist. Also, about half of the included studies were of moderate quality suggesting further research is likely to have an important impact on our confidence in the estimates. However, to our knowledge, this is the first study that provides comprehensive and contemporary evidence on the burden of cardiac valvulopathies in patients with MFS. The robustness of the search and non-application of restrictions in a large database provides valuable information needed to draw the global cartography of MFS-related structural valvulopathies.

## Conclusions

In patients with MFS, the frequency of structural cardiac valve diseases can be approximated as mitral valve prolapse at 65%; mitral valve regurgitation at 40%; aortic valve regurgitation at 40%; tricuspid valve prolapse at 35%; tricuspid valve regurgitation at 43%; and pulmonary valve prolapse at 5.3%. In this population, the occurrence of valvular stenosis is quite uncommon. Close surveillance is appropriate.
